# Anomalous origin of the left coronary artery from the right aortic sinus: probably benign variant associated with a subpulmonic intramyocardial course – a case report

**DOI:** 10.1186/s12887-020-1988-3

**Published:** 2020-03-23

**Authors:** Anja Hanser, Andreas Hornung, Ludger Sieverding, Jürgen Schäfer, Michael Hofbeck

**Affiliations:** 1 Department of Pediatric Cardiology, University Children’s Hospital, University of Tübingen, Hoppe-Seyler-Str. 1, 72076 Tübingen, Germany; 2 Department of Diagnostic and Interventional Radiology, University Hospital of Tübingen, Hoppe-Seyler-Str. 3, Tübingen, 72076 Germany

**Keywords:** Anomalous aortic origin of the left coronary artery, Subpulmonic intramyocardial course, Congenital heart disease, Sudden cardiac death

## Abstract

**Background:**

Anomalous aortic origin of the left coronary artery (AAOLCA) from the right aortic sinus is a rare congenital anomaly associated with significantly increased risk of myocardial ischemia, arrhythmias and sudden cardiac death. This refers specifically to AAOLCA associated with interarterial and/or intramural course. Much less is known about anomalous origin of the left coronary artery from the right aortic sinus associated with a subpulmonic intramyocardial course.

**Case presentation:**

We report a 12 year old girl who complained of recurrent episodes of chest pain lasting for some minutes and not associated to exercise. Echocardiography revealed AAOLCA from the right aortic sinus taking a subpulmonic course within the conal septum. The diagnosis was confirmed by CT-scan and selective coronary angiography. Treadmill test, Holter-ECG and bicycle-stress echocardiography revealed no evidence of myocardial ischemia reflecting unimpaired diastolic flow in the left coronary artery. According to the nature of the complaints and in the absence of signs of myocardial ischemia the episodes of chest pain were classified as idiopathic and not associated to the coronary anomaly. We opted for a conservative approach with regular follow-up visits. During a follow-up of 2 years without restriction of sports activities the patient has been asymptomatic.

**Conclusion:**

According to the literature AAOLCA with subpulmonary intramyocardial course appears to be associated with significantly less clinical problems than AAOLCA taking an interarterial course. The diagnosis can be suspected based on echocardiography and confirmed by contrast-enhanced computed tomography. Until now there are only few data concerning the natural history and incidence of complications in this specific anomaly. Despite the probably benign nature we recommend regular follow-up examinations including stress-testing in these patients.

## Background

Anomalous aortic origin of a coronary artery is a rare congenital anomaly with a prevalence estimated between 0.1–0.7% [1]. The ratio of anomalous right coronary to anomalous left coronary artery is reported three-to-one [1]. Anomalous aortic origin of the left coronary artery (AAOLCA) from the right aortic sinus is associated with significantly increased risk of myocardial ischemia, life-threatening arrhythmias and sudden cardiac death, especially if AAOLCA is associated with interarterial and/or intramural course [2, 3]. The AAOLCA can also take a course anterior to the right ventricular outflow tract (prepulmonic) or posterior to the aorta (retroaortic). These latter variants are generally considered benign although rare case reports of ischemia have been reported [2, 4, 5]. Much less is known about anomalous origin of the left coronary artery from the right aortic sinus associated with a subpulmonic intramyocardial course.

## Case presentation

**Fig. 1:**
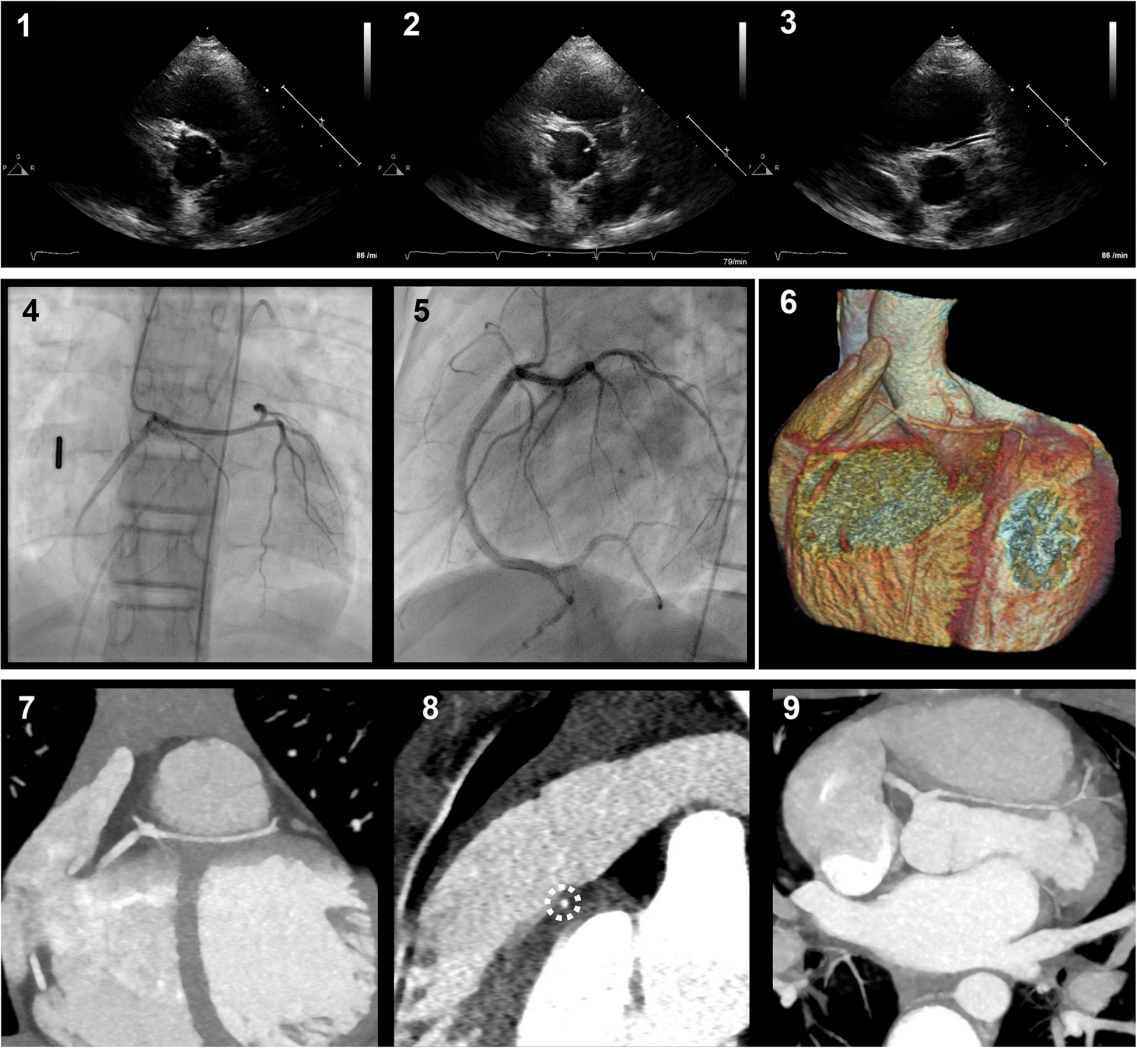
**1**-**3** Echocardiography in the parasternal short axis view, showing origin of both coronary arteries from the right aortic sinus; **4-5** Selective coronary angiography confirming AAOLCA from the right aortic sinus; **6** Cardiac CT-scan with prospective gating, in coronal plane showing AAOLCA from the right aortic sinus taking a subpulmonic intramyocardial course, volume rendering technique (VRT), AW-Server 2.0; GE, General Electric Company, Boston (USA); **7-9** Cardiac CT-scan in the coronal, sagittal and transversal plane showing AAOLCA from the right aortic sinus taking a subpulmonic intramyocardial course (circle) below the right ventricular outflow tract (syngo.via; Siemens Healthcare GmbH; Erlangen, Germany)


We report a 12 year old girl, who first presented at the age of 8 years for evaluation of recurrent episodes of chest pain not associated with exercise and lasting for several minutes. Echocardiography showed AAOLCA from the right aortic sinus (Fig. 1 1-3). The coronary artery took a caudal course below the right ventricular outflow tract. In the absence of signs of myocardial ischemia and since the episodes of chest pain had the characteristics of idiopathic chest pain [6] we decided to monitor the patient in the first decade without invasive diagnosis. When she was referred again at the age of 12 years she still had short episodes of chest pain but otherwise she was asymptomatic. To exclude possible myocardial ischemia in the presence of the coronary anomaly we now proposed a full cardiac work up. Cardiac MRI showed normal cardiac function. Treadmill testing revealed good exercise capability without signs of myocardial ischemia while bicycle-stress echocardiography showed normal left ventricular function without any regional wall motion abnormalities. 48-h Holter-monitoring showed sinus rhythm without any arrhythmias. Selective coronary angiography, performed under deep intravenous sedation, confirmed origin of the left coronary artery from the right aortic sinus (Fig. 1 4-5). The left coronary artery did not take an interarterial but rather an inferior, subpulmonic course. This was further clarified by contrast-enhanced computed tomography (Somatom Force, Siemens) which clearly demonstrated the intramuscular nature of this coronary arterial course (Fig. 1 6-9, video). Furthermore the CT-scan revealed that the anomalous left coronary artery had neither a slit like opening nor an acute angle of take-off from the aorta which have been identified as possible risk factors for myocardial ischemia in patients with interarterial course [7]. Since the episodes of chest pain had the characteristics of idiopathic chest pain [6] and since prophylactic surgery appeared not justified in the absence of documented ischemia we opted for a conservative approach without restriction of exercise or sports activities and recommended annual follow-up visits [2]. During a follow-up of 2 years the patient remained asymptomatic.

**Additional file 1: Video.** Cardiac CT-scan with prospective gating, in coronal plane showing AAOLCA from the right aortic sinus taking a subpulmonic intramyocardial course, volume rendering technique (VRT), AW-Server 2.0; GE, General Electric Company, Boston (USA)


## Discussion

There are few reports and studies in the literature describing AAOLCA taking a subpulmonic intramyocardial course to the left ventricle [8]. In the large series of Cheezum et al. the prevalence of this anomaly was higher than the respective anomaly associated with an interarterial course: Among 5991 patients undergoing coronary computed tomographic angiography anomalous origin of a coronary artery arising from the opposite sinus was detected in 103 patients involving 110 coronary arteries. AAOLCA with subpulmonary course was found in 8/110 coronary arteries as compared to 2/110 cases of AAOLCA associated with an interarterial course [9]. Although in AAOLCA with subpulmonic course the LCA is subject of potential myocardial compression during systole, this anomaly appears to be associated with less clinical problems than AAOLCA in patients with an interarterial course [10]. Therefore AAOLCA with subpulmonic course has been addressed as generally benign [1, 2, 7] and only exceptional cases requiring surgery due to myocardial ischemia have been reported in two recent series [9, 11]. According to the experience in our patient the diagnosis of this anomaly can be suspected by echocardiography and confirmed by contrast enhanced computed tomography. Selective coronary angiography was helpful to exclude peripheral compression of the left coronary artery. To exclude myocardial ischemia the AATS expert consensus guidelines recommend to perform exercise stress testing and stress echocardiography or exercise stress testing combined with nuclear perfusion scan [2].

Until now there are only few data on the natural history and long term outcome of this anomaly. Further data based on large registries will be required to clarify this question. If surgery is required in symptomatic patients with AAOLCA and subpulmonic course the focus is to abolish potential compression of the intraconal portion of the left coronary artery. This can be achieved by division of muscle fibers overlying the coronary artery and by anterior translocation of the pulmonary artery using a modified LeCompte manoeuvre [12]. This approach represents an extensive surgical procedure and it should be reserved to symptomatic patients. Since our patient never presented signs of myocardial ischemia we decided not to restrict physical exercise and to follow her with annual follow-up visits including ECG, echocardiography and exercise stress testing [2, 7].

## Conclusion

In our case report we describe a rare variant of AAOLCA from the right aortic sinus. Because of its subpulmonic intramyocardial course it has to be discriminated from variants taking an interarterial and intramural course. Although the subpulmonic course has been reported as a benign variant we suggest to perform regular cardiologic surveillance of these patients with annual follow-up visits to create robust data on the prognosis of this rare anomaly.

## Data Availability

The datasets used and /or analysed during the current study available from the corresponding author on reasonable request.

## Abbreviations

AAOLCA: Anomalous aortic origin of the left coronary artery
LCA: Left coronary artery
